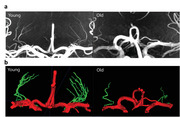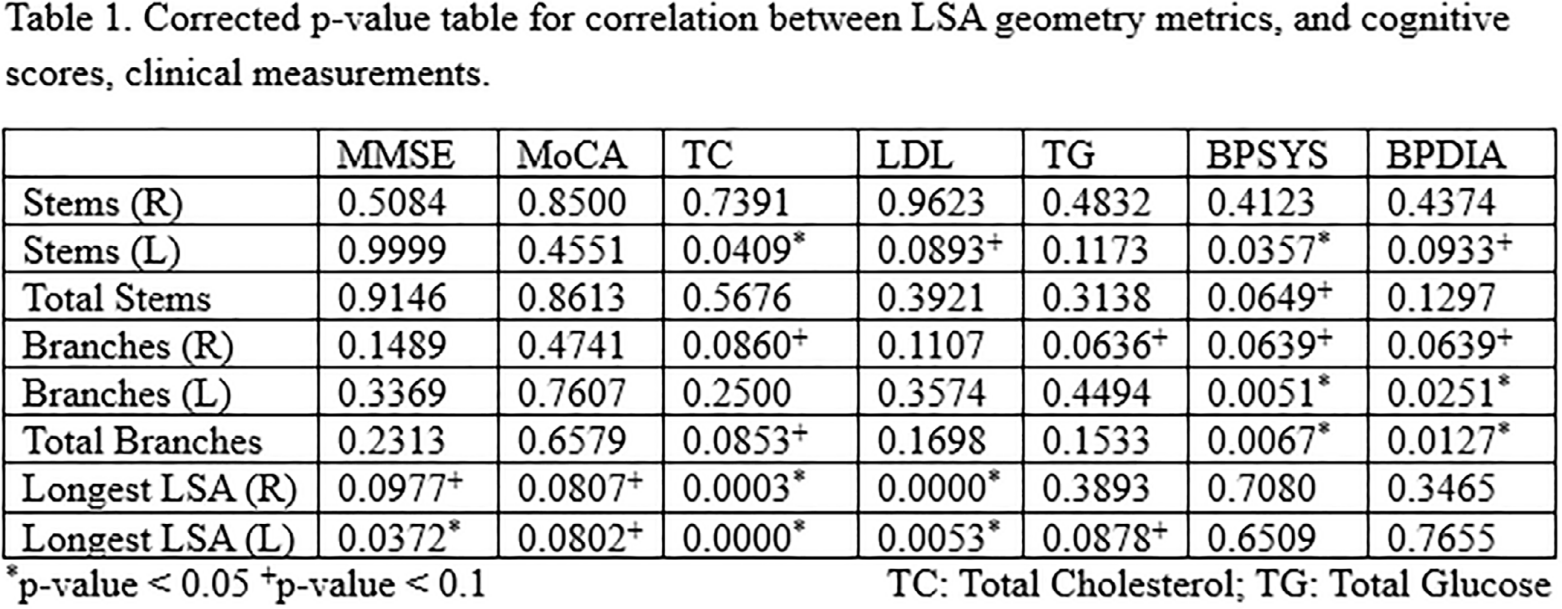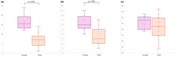# Age‐Related Morphological Changes in Lenticulostriate Artery using 7T Time‐Of‐Flight MRI: Associations with Cognitive Function and Vascular Risk Factors

**DOI:** 10.1002/alz70856_097369

**Published:** 2025-12-24

**Authors:** Tarin T. Tanji, Michael Anukwu, Jianing Tang, Maria T. Gamez, Lirong Yan

**Affiliations:** ^1^ Rush University Medical College, Chicago, IL, USA; ^2^ Meharry Medical College, Nashville, TN, USA; ^3^ Northwestern University, Chicago, IL, USA

## Abstract

**Background:**

Lenticulostriate Arteries (LSA) are small perforating vessels originating from the middle cerebral artery (MCA), playing a critical role in the pathophysiology of cerebral small vessel disease and cognitive function.^1,2^ We aimed to analyze age‐related morphological changes in LSAs utilizing 7T high‐resolution Time‐Of‐Flight (TOF) Magnetic Resonance Angiography.

**Methods:**

Twenty‐nine participants (8 young, 23.0 ± 1.5 yrs; 21 older, 72.0 ± 9.4 yrs) were enrolled and underwent cognitive and vascular assessment. High‐resolution 3D TOF was acquired on a 7T MRI Scanner (Siemens Healthineers) with FOV = 158 x 200 x 75 mm^3^, voxel size = 0.2 x 0.2 x 0.4 mm^3^, TR/TE = 12/4.67 ms, flip angle = 17°, and time = 8 min. LSAs were manually segmented using 3D Slicer and ITK‐SNAP. LSA stems (directly connected to the MCA) and branches (daughter vessels arising from stems) were analyzed. Longest LSA lengths were determined using the centerline. Two‐sample t‐tests were used for group comparison. A linear mixed‐effect model was used for associations between LSA geometry and clinical tests, adjusting for age, gender, and education.

**Results:**

Older participants had significantly fewer LSA stems and branches than younger participants (*p* < 0.05), indicating an aging effect on LSA morphology (Figure 2). The longest LSA was shorter in older participants, though not significantly (*p* = 0.11). In older participants (Table 1), the number of LSA branches was correlated with both systolic and diastolic BP (*p* = 0.007 and 0.013), while the number of stems showed a negative trend with systolic BP (*p* = 0.07) and diastolic BP (*p* = 0.13). The longest LSA length was associated with cognitive function (*p* = 0.04 for MMSE, *p* =  0.08 for MoCA), and with total cholesterol (*p* < 0.001) and LDL cholesterol levels (*p* = 0.005).

**Conclusion:**

These results aligned with another study showing age‐related deterioration in LSA geometry.^1^ This study further demonstrated that age‐related changes in LSA geometry are closely associated with cognitive decline and vascular risk factors, including hyperlipidemia and hypertension.

**References**

1. Wei et al., 2022

2. Xu et al., 2021